# Exosomes in the Oncobiology, Diagnosis, and Therapy of Hepatic Carcinoma: A New Player of an Old Game

**DOI:** 10.1155/2018/2747461

**Published:** 2018-07-25

**Authors:** Fang Sun, Jin-Zhi Wang, Ji-Jun Luo, Yu-Qin Wang, Qin Pan

**Affiliations:** Department of Gastroenterology, Xinhua Hospital, Shanghai Jiaotong University School of Medicine, Shanghai 200092, China

## Abstract

Exosomes are emerging as essential vehicles mediated cross-talk between different types of cells in tumor microenvironment. The extensive exploration of exosomes in hepatocellular carcinoma (HCC) enhances our comprehension of cancer biology referring to tumor growth, metastasis, immune evasion, and chemoresistance. Besides, the versatile roles of exosomes provide reasonable explanations for the propensity for liver metastasis of gastric cancer, pancreatic ductal adenocarcinoma, breast cancer, and colorectal cancer. The selective-enriched components, especially some specific proteins and noncoding RNAs in exosomes, have great potential as noninvasive biomarkers of HCC with high sensitivity and specificity. The characteristics of exosomes further inspire frontier research to interrupt intercellular malignant signals by controlling the biogenesis, release, or contents of exosomes.

## 1. Introduction

Hepatocellular carcinoma (HCC), which mainly develops in the context of chronic inflammation and liver fibrosis [1], is of great concern worldwide because of its high morbidity and mortality rates [2, 3]. Unresectable tumors caused by delayed diagnosis generally develop innate or acquired chemoresistance [4]. The multifocal tumors composed of heterogeneous subpopulations, with multiple dysregulated signaling pathways, limit the efficacy of targeted therapies [5].

Studies reveal that every step of tumorigenesis and development of HCC depends on the intricate interactions with the tumor microenvironment, which comprises fibroblasts, endothelial cells, cancer stem cells, myeloid cells, and the associated soluble cytokines [6]. It has emerged that exosomes serve as crucial regulator of the tumor microenvironment by promoting HCC onset and metastasis. For example, tumor-derived exosomes carry regulatory molecules and tumor antigens that are beneficial for the survival of cancer cells and the development of the malignant phenotype. Exosomes derived from cancer-associated fibroblasts (CAFs) show a synergetic effect with cancer cells in optimizing the tumor microenvironment. In contrast, modified exosomes have been demonstrated as a promising approach to cancer treatment, whether derived from human umbilical cord, bone marrow, adipose tissue mesenchymal stem cells (MSCs), or dendritic cells.

Except for the occurrence of HCC, liver occupies a pivotal position for the metastatic organotropism of gastrointestinal cancers [7]. Organ-specific metastasis theories used to put emphasis on the intrinsic properties of cancer cells, such as breast cancer cells with chemokine receptors C-X-C motif receptor 4 (CXCR4) and C-C motif receptor 7 (CCR7), prefer the metastatic destination expressing CXCL12 (lymph nodes) and CCL21 (lung) [8]. Nowadays, tumor-derived exosomes have been proved to be critical for a well-prepared premetastatic niche [9]. The exosomal compositions vary from cells of different phenotypes and status under physiological or pathological conditions. Databases of Vesiclepedia [10], EVpedia [11], and Exocarta [12] have been established to describe exosomes and their corresponding methodology.

In this review, we summarize the multifaceted roles of exosomes in the tumor microenvironment in HCC and liver metastasis. The potential utility of exosomes as noninvasive biomarkers and in therapy for HCC is also discussed.

## 2. Exosomal Biology: Characteristics, Biogenesis, Excretion, and Integration

According to the consensus of International Society for Extracellular Vesicles (ISEV), extracellular vesicle (EV) serves as an umbrella term for secreted vesicles existing in the extracellular space, including exosome, microvesicle (MV), dexosome, tolerosomes, oncosome, and prostasome [29]. In the present review, exosomes among these ones are subjected to summarization for its biology and functions in hepatic carcinoma.

Exosomes, the 40–100 nm, rounded extracellular vesicles with lipid bilayer membrane [30], are first discovered to transport the transferrin receptor into intercellular space during the maturation of sheep reticulocytes in 1980s [31]. Nowadays, sequential ultracentrifugation method is widely applied to isolate the exosomes from body fluids or cell culture media [32]. The morphology of isolated exosomes is then identified by transmission electron microscopy (TEM), while their size distribution can be detected by nanoparticle tracking analysis (NTA) or dynamic light scattering (DLS). Furthermore, both western blot and flow cytometry reveal the markers specific to exosomes (*e.g*., CD9, CD63, and TSG101) [33].

In spite of their dynamic contents, exosomes are enriched in certain common proteins, such as tetraspanins (CD9, CD63, and CD81), membrane trafficking proteins (Rab proteins, ARF GTPases, and annexins), multivesicular body (MVB) formation-related proteins (ALIX, TSG101, and clathrin), heat shock proteins (HSP60, HSP70, and HSP90), and cytoskeletal proteins (actin and tubulin) [34]. Exceptionally, dendritic cell-derived exosomes are characterized by major histocompatibility complex class I (MHC-I), class II (MHC-II), costimulatory molecules (*i.e.*, intercellular adhesion molecule 1 (ICAM-1)), and milk fat globule EGF factor 8 (MFG-E8), which engage in docking to recipient cells [35]. Moreover, HCV-infected human hepatoma cells (Huh7.5.1) produce exosomes containing complete HCV genomes, viral proteins, and particles. These exosomes are partially resistant to neutralizing antibodies and able to transmit infection to naïve Huh7.5.1 cells [36].

Unlike microvesicles (100–1000 nm), which are directly shed from plasma membranes [37], exosomes are originated from the intracellular MVBs. Early endosomes form by inward budding of the plasma membrane. Further invagination of the endosomal membrane, mainly initiated and driven by the endosomal sorting complexes required for transport- (ESCRTs-) dependent pathway, leads to the generation of MVBs [38]. This multiprotein complex includes ESCRT-0, -I, -II, and -III, together with accessory proteins (ALG-2 interacting protein X (ALIX), vacuolar protein sorting 4 (VPS4), and vesicle trafficking 1 (VTA1)) [39]. Mechanisms underlying these processes involve recognizing ubiquitinylated proteins, recruiting deubiquitinating enzymes, and sorting specific cargos into intraluminal vesicles (ILVs) in turn. The heterogeneous cargo, containing cytosolic proteins, nucleic acids, and lipids, can be selectively incorporated into ILVs [40]. Independent of ESCRT, a variety of small RNAs, instead of 18S and 28S ribosomal RNA, enrich in the Hep3B- or PLC/PRF/5-derived exosomes in a ceramide-dependent manner [40]. Intermediated MVBs either fuse with the lysosome for degradation or fuse with the cellular membrane to form exosomes [41]. Certain Rab GTPases serving as regulators of membrane trafficking, such as RAB11 [42]/RAB35 [43] or RAB27A/RAB27B [44], facilitate the release of exosomes harboring specific cargos. Additionally, other mechanisms have been proposed for the secretion of exosomes, such as the vesicle-associated membrane protein 7- (VAMP7-) [45], negative regulator-diacylglycerol kinase *α*- (DGK*α*-) [46], and PH-dependent processes [47]. Recipient cells interact with exosomes in a stepwise manner, comprising fusion with the cell membrane, endocytosis and receptor-ligand mediated recognition, and internalization, allowing the release of the exosomal content into the cytoplasm of recipient cells [48, 49].

## 3. Exosomes Underlie Primary HCC: An Essential Role for Intercellular Communication

**Figure 1:**
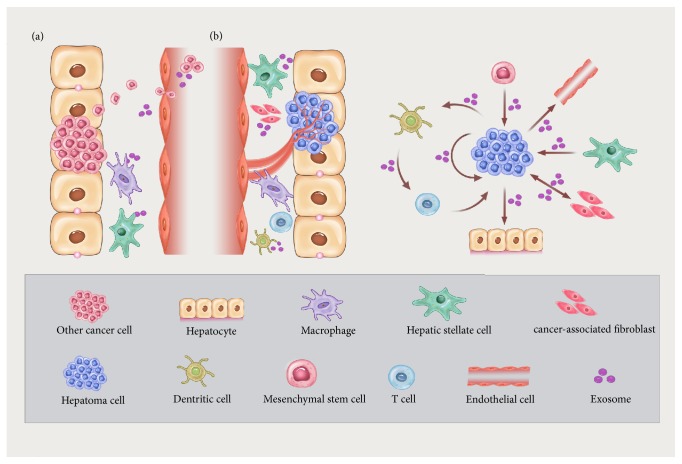
Exosomes exert regulatory effect on microenvironment by mediating the interaction of hepatic carcinoma cells and different types of liver cells. (a) Exosomes derived from tumors (*e.g.*, gastric cancer, pancreatic ductal adenocarcinoma, breast cancer, and colorectal cancer) stimulate the macrophages (Kupffer cells) and hepatic stellate cells (HSCs) to facilitate a premetastatic niche in the liver. (b) Exosomes mediate the interplay between hepatocellular carcinoma (HCC) cells and different types of liver cells, including endothelial cells (EC), cancer-associated fibroblasts (CAFs), HSCs, mesenchymal stem cells (MSCs), and dendritic cells (DCs). In detail, HCC cells promote the EC-based vascularization, HSC activation, and hepatocyte malignization by exosomes. Exosomes from MSCs, HSCs, and CAFs inhibit the malignant phenotypes of HCC cells. Moreover, DCs stimulated by HCC cells-derived exosomes are involved in the T cell infiltration and activation in HCC.

Exosomes contribute to each aspect of the interaction between intrahepatic cells, including CAFs, Kupffer cells, hepatic stellate cells (HSCs), endothelial cells, infiltrating immune cells, recruited MSCs, and cancer cells (Figure 1(b)).

### 3.1. HCC Cells

As is well known, HCC is composed of different heterogeneous subpopulations [5]. It is unclear how these tumor cells with diverse gene products closely connect with. Three metastatic HCC cell lines (HKCI-C3, HKCI-8, and MHCC-97L) shuttle protumorigenic molecules (*i.e.*, MET protooncogene, S100 family member, and caveolins), both mRNA and proteins, into hepatocyte line (MIHA)* via* exosomes. Internalization of these exosomes activates PI3K/AKT and MAPK signaling and then promotes the migratory and invasive properties of MIHAs, which resembles the donor cells of exosomes [13]. In addition, exosomal miR-122 transferred from Huh7 to HepG2 affects the expression of miR-122-regulated genes in recipient cells. IGF-1-containing exosomes derived from HepG2 cells decrease the miR-122 level in Huh7 cells reciprocally [14]. A counteracting strategy is suggested to protect HepG2 against the exogenous miR-122 from neighboring cells, thus optimizing its microenvironment to survive and develop [14]. Furthermore, transforming growth factor-*β* activated kinase-1 (TAK1) has been implicated as a central target of recipient Hep3B cells in response to a set of miRNAs (*i.e.,* miR-584, miR-517c, and miR-378) that are accumulated in Hep3B-derived exosomes [40]. TAK1, a MAP3K family member, functions as a gatekeeper for cell death and carcinogenesis of hepatocytes [50]. Exosome-mediated intercellular epigenetic modulation by miRNA resultantly trigger the loss of TAK1 with a purpose of sustaining the development of HCC [40]. Super-SILAC-based mass spectroscopy (MS) analyses have been conducted to compare the differentially expressed proteins of exosomes between nonmotile Hep3B cells and the motile MHCC-97H and LM3 cells. The glycolytic enzyme of GADPH, together with hypoxia-associated proteins caveolin-1 and calpain 1 (CAPN1), demonstrates remarkable upregulation in motile HCC cell-derived exosomes [51]. Mechanically, HCC cells under hypoxic conditions are prone to choose glycolysis as energy supply instead of mitochondrial oxidation. They acquire high motility and generate related-regulatory proteins in response to the hypoxia and related metabolic alteration [52].

### 3.2. Endothelial Cells

During tumor progression, neovasculature underlies the consumption of nutrients and oxygen, as well as the transportation of metabolic wastes and carbon dioxide, of cancer cells. Accordingly, it is essential to figure out the regulation mechanisms that activate the “angiogenic switch” so as to suppress tumor metastasis [53]. Exosomes have been reported to act as carrier of vasorin (VASN) transferred from HepG2 to human umbilical vein endothelial cells (HUVECs)* via* heparin sulfate proteoglycans- (HSPGs-) mediated endocytosis. Increased migration, but not proliferation, is observed in HUVECs after their uptake of VASN-containing exosomes [15]. Additionally, cancer stem cell-like CD90^+^ Huh7 cells, which express high levels of long noncoding RNA (lncRNA) H19, influence the endothelial phenotype through the release of exosomes. Compared with the effects of Huh7-secreted exosomes (Huh7-exo), HUVECs treated with exosomes from CD90^+^ Huh7 (CD90^+^ Huh7 exo) exhibit higher expression of VEGF/VEGF-R1, tubular-like structure, and increased cell adhesion [16]. Given the angiogenic effects related to VEGF/VEGF-R1 signaling, the exosome-induced VEGF/VEGF-R1 expression, and the conversion of endothelial cell phenotype seems to be a prerequisite for intravasation or extravasation of metastatic cells [53].

### 3.3. Stromal Cells

Most cases of HCC arise in desmoplastic context; therefore, it is vital to seek effective approaches to disrupt the symbiotic cancer cell-CAF environment. Studies show that CAFs are conducive to tumor metastasis by remodeling the extracellular matrix (ECM) through secreting matrix metalloproteinases (MMPs) or by enhancing vascular mimicry formation of HCC cells through producing TGF-*β* and stromal cell-derived factor 1 (SDF1) [54, 55]. Except for the ECM remodeling by cytokines, CAFs play beneficial roles in HCC tumorigenesis and development through exosomes-based delivery of miRNAs. miR-320 is distinctly downregulated in HCC cells, CAFs, and para-cancer fibroblasts (PAFs) upon activation with TGF-*β*. Consistently, RNA sequencing indicates the lowered miR-320a level in CAF-derived exosomes in comparison to that of PAFs-derived ones [17]. Given the ameliorative effect of miR-320a against tumor phenotype by targeting PBX3, limited introduction of exogenous miR-320a in HCC might be involved in tumorigenesis [17].

By contrast, exosomal miR-1247-3p from high-metastatic HCC cells could convert normal fibroblasts into CAFs by suppressing *β*-1, 4-galactosyltransferases III (B4GALT3). The activation of *β*1-integrin-NF-*κ*B signaling in fibroblasts enhances exosomal secretion of IL-6 and IL-8, promoting tumor stemness, epithelial-mesenchymal transition (EMT), and chemoresistance in liver cancer [18]. Besides, high serum level of exosomal miR-1247-3p positively correlates to lung metastasis in HCC patients [18]. Among the stromal cells, human hepatic stellate cells (LX2) have been revealed to load tumor suppressor-miR-335 into HCC-targeting exosomes. miR-335-5p-containing exosomes lead to the repression of HCC proliferation and invasion* in vitro*, and the shrinkage of HCC tumors* in vivo* [19].

### 3.4. Immune Cells

HCC is an intractable challenge because of the absence of potent immune response. However, tumor cell-derived exosomes (TEXs), which carry multiply antigens (*i.e.*, AFP, glypican 3, and HSP-70), trigger a dendritic cells- (DCs-) mediated immune response much stronger than that of cell lysates [20]. The tumor immune microenvironment is also significantly improved in orthotopic HCC mice, manifested by increased CD8^+^ T lymphocytes infiltration, elevated levels of interferon-*γ* (IFN-*γ*), and decreased levels of interleukin-10 (IL-10) and TGF-*β* [20]. Therefore, TEXs-pulsed DC-based immunotherapy might boost the antigen presenting ability and activate cellular immunity sufficiently to counteract the immunotolerance.

## 4. Exosomes Precede Metastatic Hepatic Carcinoma: Creation of a Malignant Niche

Metastasis is responsible for a large majority of cancer-related death [56] and reflects a succession of biological events, involving local invasion, intravasation, extravasation, and colonization [53]. Accumulating evidences suggest that tumor-derived exosomes are pioneers that prepare the premetastatic niche, which is created prior to the arrival of disseminating tumor cells, providing a “favorable soil” for the seeding of tumor cells in host tissue [57] (Figure 1(a)).

### 4.1. Gastric Cancer

Investigating the liver metastasis of gastric cancer, Zhang* et al.* show that exosomes are capable of transporting membrane receptor-epidermal growth factor receptor (EGFR) into liver stromal cells, such as Kupffer cells and HSCs. This translocation of EGFR promotes the upregulation of hepatic growth factor (HGF) in stromal cells, mainly by suppressing miR-26a/b. As a result, the activation of HGF receptor c-MET results in favorable conditions for organ-specific metastasis [21]. EGFR-containing exosomes, therefore, determine the liver-specific metastasis of gastric cancer, based on microenvironment remodeling.

### 4.2. Pancreatic Ductal Adenocarcinoma (PDAC)

Integration of PDACs-derived exosomes and Kupffer cells (KCs) has been proven to induce a proinflammatory and profibrotic microenvironment in the liver. Notably, macrophage migration inhibitory factor (MIF), which is enriched in PDAC-derived exosomes, causes the secretion of TGF-*β*, which, in turn, leads to the activation of HSCs and ECM remodeling. Ultimately, fibronectin deposition promotes the recruitment of bone marrow-derived macrophages to the liver, producing a favorable niche for the liver metastasis of PDACs [22].

### 4.3. Breast Cancer

Consistent with the “seed and soil” hypothesis [58], exosomes from liver-tropic breast and pancreatic cancer cells have been found to preferentially assemble in predictive metastatic sites. The specific integrin expression pattern of tumor-derived exosomes has already been shown to govern the future colonization by tumor cells [23]. In detail, integrin subtypes of *α*6*β*4 and *α*6*β*1 in these exosomes exhibit a close association with lung metastasis by targeting fibroblasts and epithelial cells. Accordingly, exosomal integrin *α*v*β*5 determines the formation of premetastatic niche in the liver through binding to KCs, accompanied by the upregulated expression of proinflammatory gene*-S100P and S100A8 *of KCs [23].

### 4.4. Colorectal Cancer (CRC)

After the injection of exosomes collected from a colorectal cancer cell line (HT-29) with liver-targeted metastatic properties, the mice implanted with Caco-2 cells (low metastatic colorectal cancer cell line) show an increase in the hepatic burden of metastasis* via* the SDF-1*α*/CXCR4 axis [59]. Hence, considering the distinct functional components within exosomes in colorectal cancer cells with different metastatic properties, specific exosomal miRNAs may serve as indicator of the liver metastasis of colorectal cancer. Although the expression level of miR-203 in tumor tissues is negatively correlated with metastatic potential [60], high expression of serum exosomal miR-203 in patients with CRC predicts poor prognosis and liver metastasis. Exosomal miR-203 from CRC cells could induce the formation of M2 tumor-associated macrophages (TAMs) in the host organ, which constitute a premetastatic niche and promote liver metastasis [24]. In contrast to exosomes isolated from naïve colon tissue and primary colon tumors, exosomes originated from liver metastasis of colon tumors display higher levels of tumor-suppressive miRNAs. In primary colon tumor and liver metastasis of colon tumor, tumor-suppressive miRNAs (miR-18a and miR-193a) are enriched in exosomes rather than their parent cells. Selective sorting protein-major vault protein (MVP), which binds to miR-193a, might induce miR-193a accumulation in exosomes instead of in their donor cells [61]. Interestingly, oncogenic miR-21 shows more enrichment in the primary colon tumor tissue and metastatic colon tumor in the liver as compared to their exosomes [61]. These studies indicate that colon cancer may discharge those miRNAs, which have adverse effects on its progression and liver metastasis, into serum* via* exosomes.

## 5. Exosomes Identify HCC: Biomarkers for Noninvasive Diagnosis and Follow-Up Monitoring

The characteristics of exosomes represent appealing and promising biomarkers for the diagnosis and monitoring of cancer. (1) Exosome contents reflect the real-time status of their originated cells with high sensitivity. (2) Exosomes are capable of keeping their cargos intact because of their stable structure, which is resistant to degradation by circulating ubiquitous RNases and proteases. (3) Exosomes are shed into biological fluid, thus providing a noninvasive method to identify tumor-bearing patients. (4) High-enrichment of tumor markers in exosomes compared with their low baselines in body fluids increases the signal-to-noise ratio upon analysis [62].

To select serum exosomal miRNAs as potential biomarkers, Sohn* et al.* report that exosomal levels of miR-18a, miR-221, miR-222, and miR-224 are higher in patients with HCC than in those with chronic hepatitis (CHB) or liver cirrhosis (LC). However, exosomal miR-101, miR-106b, miR-122, and miR-195 show the reverse tendency [63]. Microassay profiling in cases of HCC identifies that decreased miR-718 level in serum exosomes is positively correlated with recurrence following liver transplantation (LT) and poor prognosis of patients with HCC [64]. Despite miRNAs (miR-423-5p and miR-21-5p) being enriched in both cellular and exosomal compositions of SMCC-7721 (HCC cell line), disparities in the miRNA pattern between exosomes and their donor cells still exist. While highly abundant let-7d-5p, let-7b-5p, and let-7c-5p are observed in exosomes rather than cells, miR-486-5p and miR-10b-5p are elevated within cells only [65]. This implies that sequestering miRNAs in exosomes is an active biological process [65]. In addition to dysregulated miRNAs, increased lncRNA TUC339 is also detected in HCC-derived exosomes and is positively correlated with proliferation and cell adhesion of HCC [66].

Recent study analyzes proteome profiles of serum exosomes isolated from cholangiocarcinoma (CCA), primary sclerosing cholangitis (PSC), patients with HCC, and healthy individuals. A variety of differentially expressed proteomic signatures are determined. Galectin-3-binding protein (LG3BP) and polymeric immune receptor (PIGR) in serum exosomes show better diagnostic value for HCC (area under the curve (AUC): LG3BP = 0.904 and PIGR = 0.837, respectively) compared with that of alpha fetoprotein (AFP) (AUC: 0.802), which is most commonly used as a noninvasive serum indicator of HCC. However, PIGR appears to be a nonspecific, inflammation-related marker. Moreover, the high levels of exosomal pantetheinase (VNN1), C-reactive protein (CRP), fibrinogen gamma chain (FIBG), immunoglobulin heavy constant alpha 1 (IGHA1), alpha-1-acid glycoprotein 1 (A1AG1), and gamma-glutamyltransferase 1 (GGT1) in CCA might benefit the differential diagnosis of intrahepatic CCA (iCCA) versus HCC [67]. Given the complexity of tracking the origin of molecular signatures and the lack of standardization of assessment, further efforts are needed to replace traditional biomarkers with exosome-based biomarkers.

## 6. Exosomes Cure HCC: Therapeutic Application of Exosomal Modification

### 6.1. Modulation of Exosomal Excretion

Emerging evidence identifies an essential mechanism of preserving cellular homeostasis in presenescent cells by loading harmful cytoplasmic DNA fragments into exosomes under physiological conditions [68]. Likewise, the selective release of specific molecules into exosomes has a fundamental impact on their parental cells during tumor progression.

Silencing of RAB27A or RAB27B in bladder carcinoma prevents the discarding of tumor-suppressive miR-23b and miR-921 into exosomes, which affect the acquisition of metastatic properties [69]. Vps4A, which is frequently downregulated in HCC, has been proven to be a crucial regulator of exosomes biogenesis by targeting ESCRT-III [70]. Restoration of Vps4A alters the secretion and uptake of exosomal miRNAs in HCC cells. In brief, SMCC-7721 cells transfected with Vps4A tend to package oncogenic miRNAs (miR-27b-3p and miR-92a-3p) into exosomes and accumulate tumor suppressor miRNAs (miR-193a-3p, miR-320a, and miR-132-3p) inside the cells. Being compared to those in the SMCC cells coincubated with Vps4A-lacking exosomes (SMCC-ctrl-exo), five tumor-suppressive miRNAs (miR-122-5p, miR-33a-5p, miR-34a-5p, miR-193a-3p, miR-16-5p, and miR-29b-3p) are proved to be upregulated in Vps4A-overexpressing SMCCs exposure to SMCC-ctrl-exo [65]. In contrast to the Vps4A-dependent exosome-mediated interchange of specific miRNAs, inhibition of exosome secretion provokes the reactive oxygen species- (ROS-) dependent DNA damage response and then triggers senescence-like, cell-cycle arrest or apoptosis [68].

### 6.2. Immunotherapy

Rao* et al.* [20] have demonstrated that HCC-derived exosomes, which could serve as carrier of multiple antigens, trigger a DC-mediated immune response stronger than lysates. Suppression of tumor growth has also been observed in ectopic and orthotopic HCC mice treated with this kind of TEXs-pulsed DCs. In addition, these TEXs could be a source of shared antigens for tumors of different origins. DCs pulsed with TEXs from Hepa1-6 (mouse HCC cell line) resultantly provide cross protective effects against allogeneic H22 (mouse HCC cell line) and MHC-matched pancreatic cancer cells. Moreover, tumor-specific cytolysis is observed in human HCC cells (Hep3B, LM3) treated with activated DCs by TEXs from HepG2, independent of HLA types [20].

In contrast, exosomes derived from AFP-expressing DCs (DEX_AFP_) elicit an antigen-specific antitumor immune response, especially in the diethylnitrosamine- (DEN-) induced autochthonous HCC model. Serving as a cell-free vaccine, DEX_AFP_ improve the immune-suppressive tumor milieu* via* activation of CD8^+^ cytotoxic T lymphocytes (CTLs) and elevated IFN-*γ* and IL-2 levels, but fewer CD25^+^/Foxp3^+^ regulatory T (Treg) cells and decreased IL-10 and TGF-*β* levels [71]. Thus, tumor-specific antigen-modified DEXs present a new pathway for HCC immunotherapy.

In terms of DC-mediated immunotherapy, a phase II clinic trial using exosomes derived from IFN-*γ*-matured DCs (IFN-*γ*-DEX) is being conducted in patients with advanced nonsmall-cell lung carcinoma (NSCLC) at 4 months after platinum-based chemotherapy. Benefitting from the enhanced T cell response, DEX-based maintenance immunotherapy yields improved progression-free survival. Thirty-two percent of patients who received IFN-*γ*-DEX therapy have achieved stabilization for 4 months or more. The median overall survival (OS) for all patients is 15 months, with a survival rate at 6 months of 86%, at 1 year of 55%, and at 2 years of 25% [72].

### 6.3. Chemosensitivity

Resistance to chemotherapeutic drugs is a major issue of treatment failure of cancers. And exosomes are proved to mediate the horizontal transfer of property of chemoresistance among different populations of HCC cells. Qu* et al.* show that HCC cells (MHCC-97 L and MHCC-97H) secreted exosomes prompt sorafenib resistance and inhibit sorafenib-induced apoptosis of SMCC-7721 cells* in vitro* and* in vivo* [25]. Exosomes derived from highly invasive HCC cell (MHCC-97H) have greater effect than those from the less invasive cell (MHCC-97L) [25]. The intercellular delivery of HGF, and subsequently activation of HGF/c-MET/Akt signaling, might account for the acquired sorafenib resistance [25].

Except for the intercellular transfer of drug-transporters [73], genetic cargos within tumor-derived exosomes refer to another mechanism that limits the response of target cells to anticancer drugs. Takahashi* et al.* attribute the TGF*β*-induced tumor cells dedifferentiation and acquired chemoresistance to the deregulated linc-ROR in HepG2 cells, whereas sorafenib exposure increases linc-ROR in HCC cells, HCC-derived exosomes, and exosome-treated recipient cells [26]. Similarly, chemotherapeutic stress (*e.g.*, sorafenib, camptothecin, and doxorubicin) leads to linc-VLDLR upregulation in both HCC cells (HepG2) and exosomes. Exosomal delivery of linc-VLDLR increases the expression of ABCG2 (ATP-binding cassette, subfamily G member 2) [27], which guarantees an inadequate concentration of the toxicant via drug export [74]. Thus, a chemoresistant property is conferred on the recipient HCC cells. In addition, the intercellular delivery of HGF and subsequently activation of HGF/c-MET/Akt signaling might account for the acquired sorafenib resistance [25]. Since the EMT-dependent deprivation of chemosensitivity [75], the role of HCC-derived exosomes in driving EMT may also be one of the determining factors in chemoresistance [76].

### 6.4. Exosomal Therapy Based on Mesenchymal Stem Cells

Exosomes possess promising properties of vehicles, including biocompatibility, inherent stability, and selective integration at the targeted site [21, 32]. They are also able to penetrate the blood-brain barrier, endure modification, and load drugs [77, 78]. Moreover, MSCs can mobilize into the tumor microenvironment [79]. MSC-secreted exosomes, therefore, are considered a prospective research hotspot for therapeutic application with feasibility and effectiveness against tumors.

Exosomes originating from human umbilical cord MSCs have been proposed to counteract 5-fluorouracil-induced apoptosis and abrogate chemosensitivity in gastric cancer. Mechanically, MSC-derived exosomes induce the activation of the CaM-Ks/Raf/MEK/ERK pathway in gastric cancer cells and further promote the expression of multidrug resistance associated proteins, such as multidrug-resistance protein (MDR), multidrug-resistance like protein 1 (MRP), and lung resistance protein (LRP) [80]. MSC-derived exosomes also facilitate angiogenesis and stimulate tumor growth when coinjected with tumor cells in nude mice [81].

When the murine MSC line (SR4987) is loaded with Paclitaxel (PTX), the PTX-MSC-derived exosomes demonstrate the capacity to attenuate the proliferation of a pancreatic cell line (CFPAC-1) [82]. Employing the Cy5-labelled tracking method, Jessian* et al.* verify the anti-miR-9 delivery from bone marrow- (BM-) MSCs to glioblastoma multiforme (GBM)* via *exosomes. Then exogenous anti-miR-9 then reverses the expression of P-glycoprotein (P-gp) and sensitizes GBM to temozolomide (TMZ) [83]. Another HCC model is constructed in Fischer-344 rats by subcutaneous inoculation with HCC cells obtained from N1S1 rats. However, treatment with exosomes, being secreted by adipose-derived mesenchymal stem cells (ADMSCs), produce smaller, lower grade tumors that harbor an increased natural killer T (NKT) cell response [84]. Another study shows that intratumoral injection of miR-122-modified ADMSC-derived exosomes, combined with sorafenib, could significantly reduce tumor volume and weight [28]. Although 122-exo could alter the expression of target genes, such as those encoding cyclin G1 (CCNG1), disintegrin and metalloprotease 10 (ADAM10), and insulin-like growth factor receptor 1 (IGF1R) in hepatoma cells, 122-exo alone failed to inhibit tumor growth in a HepG2 cell xenograft model in nude mice [28].

## 7. Conclusion

The exosome-mediated intercellular transfer of biomolecules has been demonstrated in a myriad of process associated with HCC onset, progression, and poor response to chemotherapy (Table 1). The occurrence and propensity of liver metastasis may be ascribed to exosomes derived from primary cancer cells. In addition, exosomes possess promising characteristics of biomarkers, especially through monitoring their content of oncogenic and tumor-suppressive miRNAs. Importantly, exosome-based treatment holds great prospect in cancer by disrupting tumor cell homeostasis, activating immune response, and delivering chemotherapeutic agents. Despite all these achievements, further explorations are still needed to comprehensively evaluate the clinical application of exosomal functional molecules concerning the diagnosis and therapy of liver cancer.

## Figures and Tables

**Table 1:** Exosome-mediated cellular interaction involved in liver cancer.

**Donor cells**	**Components**	**Recipient cells**	**Functions**	**References**
HKCI-C3 HKCI-8 MHCC-97L	MET proto-oncogene, caveolins, S100 family	MIHA	Migration, invasion	[13]

Huh7	miR-122	HepG2	Proliferation	[14]

HepG2	VASN	HUVECs	Migration	[15]

CD90+ Huh7	lncRNA H19	HUVECs	Angiogenesis, adhesion	[16]

CAFs	miR-320a	MHCC97-H	Proliferation, migration, metastasis	[17]

CSQT-2 HCC-LM3	miR-1247-3p	Normal fibroblasts	Conversion into CAFs	[18]

LX-2	miR-335	MHCC97H, MHCC97L, HepG2 and Huh7	Proliferation, invasion, tumor shrinkage	[19]

Hepa1-6	*α*-AFP, glypican 3, HSP 70	DCs	T cell activation	[20]

Gastric cancer	EGFR	Kupffer cells HSCs	Liver metastasis	[21]

Pancreatic ductal adenocarcinoma	MIF	Kupffer cells HSCs	Liver metastasis	[22]

Breast cancer	integrin *α*v*β*5	Kupffer cells	Liver metastasis	[23]

Colorectal cancer	miR-203	M2-TAMs	Liver metastasis	[24]

MHCC-97H	HGF	SMCC-7721	Chemoresistance	[25]

HepG2	linc-ROR	HepG2	Chemoresistance	[26]

HepG2	linc-VLDLR	HepG2	Chemoresistance	[27]

ADMSCs	miR-122	HepG2	Sorafenib sensistivity	[28]

ADMSCs: adipose-derived mesenchymal stem cells; CAFs: cancer associated fibroblasts; DCs: dendritic cells; HSCs: hepatic stellate cells; HUVECs: human umbilical vein endothelial cells; H22: murine hepatocarcinoma cell line; LX-2: hepatic stellate cells line; MIF: macrophage migration inhibitory factor; MIHA: immortalized hepatocyte cell line; and TAMs: tumor-associated macrophages.
